# Immune–inflammatory–nutritional dysfunction in HIV-negative patients with asymptomatic neurosyphilis: a retrospective comparative study

**DOI:** 10.3389/fimmu.2026.1890842

**Published:** 2026-08-17

**Authors:** Huiqin Cao, Meidi Wang, Jun Wang, Jing Han, Yongxing Yan

**Affiliations:** 1 Nursing Department, Hangzhou Third People’s Hospital, Hangzhou, Zhejiang, China; 2 Department of Neurology, Hangzhou Third People’s Hospital, Zhejiang, Hangzhou, China

**Keywords:** asymptomatic neurosyphilis, CALLY index, immunity, inflammation, nutrition, syphilis

## Abstract

**Objectives:**

The host’s immune function, inflammatory response, and nutritional status are closely related to the occurrence and development of neurosyphilis (NS). Asymptomatic neurosyphilis (ANS), as a completely curable type of NS, has received limited study on these fields. This study explores the immune function, inflammatory response, and nutritional status levels in patients with ANS, as well as their changes after treatment, aiming to provide reference for the early diagnosis and treatment of ANS.

**Methods:**

This study retrospectively included 32 healthy controls (HCs), 27 cases of ANS, and 29 cases of syphilis who were treated in our hospital from March 2020 to September 2025. We collected peripheral blood cell/humoral immunity, inflammatory response, and nutritional status indicators from the three groups, compared the differences between them, and analyzed the changes in various indicators of patients with ANS after 6 months of treatment. We used logistic regression analysis to identify independently associated factors for ANS, and constructed receiver operating characteristic (ROC) curves to evaluate the predictive value of influencing factors for its diagnosis.

**Results:**

Compared with the syphilis and HC group, the percentages of natural killer (NK) cells, IgA levels, lymphocyte counts, content of albumin and total protein, and the C-reactive protein–albumin–lymphocyte (CALLY) index in the ANS group were significantly reduced (all *p* < 0.05), However, the neutrophil counts, neutrophil/lymphocyte ratio (NLR), platelet/lymphocyte ratio (PLR), systemic immune inflammatory index (SII), and the proportion of blood toluidine red unheated serum test (TRUST) titers ≥1:16 were significantly increased (all *p* < 0.05). Multivariate logistic regression analysis showed that the low levels of the CALLY index are an independently associated factor for patients with syphilis with concurrent ANS. Its area under the curve (AUC) for predicting ANS is 0.766, with a sensitivity and specificity of 65.5% and 88.9%, respectively. Further analysis showed that the CALLY index in patients with ANS was negatively correlated with white blood cell counts and protein contents of cerebrospinal fluid (CSF) (*r* = −0.6227, *p* = 0.0005; *r* = −0.5690, *p* = 0.0020). After 6 months of treatment, the neutrophil counts, NLR, PLR, SII, CSF, white blood cell counts, and protein contents in patients with ANS were significantly decreased (all *p* < 0.01), while the percentage of NK cells and the CALLY index significantly increased (*p* < 0.05, *p* < 0.01).

**Conclusions:**

Although patients with ANS are asymptomatic in clinic, there are disturbances in immune function, inflammatory response, and nutritional status. Early standardized treatment may reverse this imbalance. The CALLY index showed preliminary discriminatory ability for the early diagnosis of ANS while it requires validation in large-sample, multicenter cohort studies.

## Introduction

Syphilis is a chronic sexually transmitted disease caused by infection with *Treponema pallidum* (*T. pallidum*). According to epidemiological data, the global number of syphilis cases has increased from 45 million in 1990 to 70 million in 2021 (1). Among them, the number of new cases among the population aged 15–49 increased from 7.1 million in 2020 to 8 million in 2022 (2). When *T. pallidum* breaks through the blood–brain barrier and infiltrates the central nervous system (CNS), it can be diagnosed as neurosyphilis (NS). NS can occur at any stage of syphilis, especially in early untreated patients (3). Ghanem et al. found that approximately 4%–10% of untreated patients with early syphilis may progress to NS (4). Although the current prevalence of NS is lower than in the pre-penicillin era (5), misuse of antibiotics in recent years may lead to inadequate treatment, which may lead to an increased prevalence in chronic and latent syphilis, which in turn promotes the development of NS. In addition, because of the tolerance of *T. pallidum* or poor drug permeability to the CNS, treatment failure may occur, leading to recurrent episodes of NS (6). This situation not only poses a challenge to the public health system, but also emphasizes the urgency of improving prevention and control strategies. Early NS usually presents as asymptomatic meningitis, and later, it may also cause symptoms such as headache, stroke, epileptic seizures, cranial nerve paralysis, dementia, and personality and behavioral changes, and it may even lead to death in some severe patients (7–9), seriously affecting the quality of life in patients and increasing the burden on families and society. However, patients with ANS can be completely cured through standardized treatment in the early stage, which highlights the importance of early diagnosis and treatment.

At present, the diagnosis of NS mainly relies on cerebrospinal fluid (CSF) testing, serological testing, and imaging techniques. However, the sensitivity and specificity of these methods are still controversial (10–12). Patients with NS typically exhibit an increase in white blood cell (WBC) counts and protein contents in CSF. The positive result of the CSF-Venereal Disease Research Laboratory (CSF-VDRL) test is usually considered the gold standard for its diagnosis. However, this test shows low sensitivity even in patients with symptomatic NS, which can easily lead to missed diagnosis (13, 14). In addition, CSF-VDRL testing may remain reactive for a long time in patients with ANS even after adequate treatment (15). In recent years, some studies found that some biomarkers of CSF, such as CXCL13 and CXCL10, have shown excellent sensitivity and specificity in the diagnosis of NS (16–18), but patients have poor compliance with lumbar puncture, especially in patients with ANS. This leads to delays in early diagnosis and treatment. Therefore, it is extremely important to search for blood biomarkers, which are easier to obtain for the early diagnosis of ANS. It is worth noting that recent research has made progress in the development of non-invasive diagnostic methods. Xie et al. (19) demonstrated that serum ubiquitin C-terminal hydrolase L1, glial fibrillary acidic protein, and neurofibrillary light chain may serve as potential biomarkers for the diagnosis of NS, providing prospects for avoiding the discomfort of lumbar puncture, improving patient’s compliance. Chen et al. (20) also found the diagnostic utility of these biomarkers in HIV-negative patients with NS, providing a promising tool for clinical diagnosis of NS. At present, the diagnostic biomarkers for NS are mostly targeted in patients with symptomatic NS, and there are mostly single indicators. Furthermore, there are relatively few similar studies about patients with ANS that can be completely curable. In the clinical real world, patients with ANS are precisely the type of patients that clinicians need to focus on.

Although the incidence of NS is gradually increasing, the specific pathogenesis has not been fully understood. Among them, the host’s immune function and inflammatory response play important roles in the pathophysiology of NS. The interaction between *T. pallidum* and host’s immunity, as well as pathogen-induced immune/inflammatory responses, may lead to tissue damage and accelerate disease progression. A significant pathological feature of NS is the strong and compartmentalized neuroimmune response in the CNS (21). Furthermore, some recent studies have shown that inflammatory cells in the peripheral blood of patients with NS cause neurological damage by disrupting the blood–brain barrier. The brain tissue of patients with NS shows significant activation of humoral and cellular immunity, as well as perivascular infiltration of various inflammatory cells (22). These studies all reflect the complex role of immune response in the pathological process of NS, suggesting that relevant immune-inflammatory biomarkers may provide assistance for the early diagnosis of ANS. Meanwhile, the patient’s nutritional status is also crucial to the onset and progression of many diseases, as changes in nutritional status can affect the functional recovery of patients (23–25). Previous studies suggest that serum biomarkers play a certain role in the diagnosis, severity, and prognosis assessment of some infectious diseases (26–29). However, in recent years, the composite index established based on multiple serological indicators has gradually attracted the attention of many scholars (30–33). Among them, the C-reactive protein–albumin–lymphocyte (CALLY) index is composed of C-reactive protein (CRP), albumin, and lymphocyte counts, which integrates three classic clinical indicators. It reflects not only the patient’s inflammatory response, but also the patient’s nutritional and immune status (34, 35). It can more comprehensively reflect the patient’s steady-state level compared to a single indicator. It is reported that the CALLY index, as a composite index of inflammation–nutrition–immunity, has been applied to the diagnosis and prognosis assessment of tumors, cardio-cerebrovascular diseases, infectious diseases, etc., and has been proven to have certain clinical value (36–39). However, as a CNS complication caused by *T. pallidum* infection, the onset of ANS involves the host’s inflammation–nutrition–immune function, and there is currently limited study on the relationship between the CALLY index and the ANS.

Therefore, this study analyzed the peripheral blood indicators reflecting the immune function, inflammatory response, and nutritional status of patients with ANS treated in our center, surveyed the changes in these parameters after treatment, and evaluated the value of the CALLY index in the diagnosis and efficacy of ANS, in order to provide reference for clinicians to early diagnosis and treatment of ANS.

## Materials and methods

### Subjects

Patients with syphilis who were consecutively admitted in our hospital from March 2020 to September 2025 were included and divided into the ANS group and the syphilis group, as well as 32 individuals who underwent physical examination in our hospital, comprising the healthy control (HC) group. This study has been approved by the Ethics Committee of the Hangzhou Third People’s Hospital, Zhejiang, China (No. 2023KA031). All procedures were conducted in accordance with the Declaration of Helsinki.

Inclusion criteria: (1) Age was over 18 years on an individual subject; (2) all patients’ clinical symptoms, signs, and auxiliary examinations meet the diagnostic criteria for syphilis (40), determined by a dermatologist specializing in sexually transmitted diseases; (3) patients had been treated with long-acting penicillin or procaine penicillin for at least one course according to the treatment guidelines, and whose toluidine red unheated serum test (TRUST) titers decreased by less than four times or increased after 6 months of treatment; (4) for patients without a history of diagnosis or treatment, information was verified through premarital, prenatal, preoperative, or inpatient examinations with an initial serum TRUST titer ≥1:16; (5) complete clinical data were obtained; (6) reinfection and other neurological disorders were excluded; (7) the diagnosis of syphilis was based on serum TRUST (+) and treponema pallidum gelatin particle agglutination test (TPPA) (+), but CSF TRUST (−) and TPPA (−), with CSF WBC < 5 × 10^6^/L and protein < 500 mg/L; and (8) the diagnostic criteria for ANS were based on our previous research standard (41) and the diagnostic criteria of Centers for Disease Control and Prevention (CDC) (42). In short, patients had no clinical symptoms. Serum and CSF tests showed positive results for TPPA and TRUST; elevated CSF protein (>500 mg/L) or CFS WBC counts (≥5 × 10^6^/L), and other potential causes of CSF abnormalities were ruled out.

Exclusion criteria: (1) Women who were breastfeeding or pregnant; (2) patients positive for HIV; (3) merge acute infections from other parts of the body within the past month; (4) patients with autoimmune diseases or malignant tumors; (5) patients with severe heart, liver, kidney, and other organ injuries; and (6) blood transfusion treatment within the past 3 months.

Inclusion and exclusion criteria for the HC group: We randomly selected age- and gender-matched individuals who came to our hospital for physical examination in the same period, had no infectious diseases in the past month (some with chronic diseases), and had normal blood biochemical examination results, with negative HIV, TPPA, and TRUST testing.

### Methods

Clinical data are collected, including general information, results of peripheral blood, and CSF parameters test.

General information includes age, gender, body mass index (BMI), comorbidities (diabetes, hypertension, and coronary heart disease), smoking (average ≥ 20 cigarettes/day, continuous ≥ 6 months), drinking (average daily alcohol consumption ≥ 100 g/day, duration ≥ 1 year), syphilis stage, previous antisyphilitic treatment, and course of disease, among others.

Peripheral blood parameters test results: Fasting venous blood was collected from all enrolled patients on the morning of the second day of admission for testing. An enzyme-linked immunosorbent assay (ELISA) method was used to detect serum TRUST and TPPA titers in patients (Addcare ELISA 600, Yantai Addcare BioTech Co., Ltd. China). A fully automated blood cell analyzer (BC-7500, Mindray, China) was used to detect levels of high-sensitivity CRP and blood routine (including WBCs, red blood cells, platelets, hemoglobin, neutrophils, and lymphocyte counts) (Mindray Company Reagent Kit, China). A BD FACS-Calibur flow cytometer (BD Company, USA; monoclonal antibodies CD16+/CD56 PE, CD19 APC, CD3FITC, CD4 PE-Cy7, CD8 APC-Cy7, and hemolysin) was used to detect the levels of lymphocyte subsets in each group, including peripheral blood T lymphocyte subset levels [CD3+, CD4+, CD8+, CD4+/CD8+, CD19+, CD56+, the percentage of T lymphocyte, B lymphocyte, helper T cell, and natural killer (NK) cell, etc.]. The humoral immune function included serum immunoglobulin (IgA, IgG, and IgM) and complement (C3 and C4) levels. The comprehensive biochemical indicators include the detection results of total protein, albumin, globulin, creatine kinase, creatinine, urea nitrogen, electrolytes, homocysteine, total cholesterol, triglycerides, low-density lipoprotein, uric acid, etc. Using the 7600-220 fully automatic biochemical analyzer (Hitachi, Japan) for testing, with the kit provided by DiaSys Co. Ltd., Germany, we strictly followed the standards of the testing kit for testing. Meanwhile, neutrophil/lymphocyte ratio (NLR), platelet/lymphocyte ratio (PLR), neutrophil/platelet ratio (NPR), systemic immune inflammatory index (SII), platelet/albumin ratio (PAR), C-reactive protein/albumin ratio (CAR), and CALLY index values were calculated using the formula: CALLY index = {Albumin (g/L) × Lymphocyte counts (×10^9^/L) ÷ CRP (mg/L) × 10}.

CSF parameters test results: All patients with syphilis completed lumbar puncture within 2 days of admission (among them, patients in the ANS group were readmitted for lumbar puncture after 6 months of treatment). CSF parameter testing included WBC counts, protein contents, glucose, chloride, lactate dehydrogenase (LDH), adenosine deaminase (ADA), TPPA, and TRUST. ELISA was also used to detect TRUST and TPPA titers in CSF, while other indicators in CSF were detected using a fully automated biochemical analyzer (Hitachi 7600-220, Japan).

### Treatment and follow-up of patients with ANS

According to our previous treatment plan for NS (41), patients with ANS were treated with a high-dose intravenous drip of penicillin (4 million U/time) every 4 h for 14 days. Subsequently, intramuscular injection of benzylpenicillin G (2.4 million U/time) was administered once a week for 3 weeks. In 6 months after the treatment, a lumbar puncture was performed again. If the WBC counts in the CSF increases or did not decrease, or the TRUST titers in CSF increased by four times or more, high-dose penicillin would be used to treat again.

### Statistical analysis

We used SPSS27.0 software according to the stepwise structured approach for statistical analysis (43). The Shapiro–Wilk test was used to test the normality of each group of data. Measurement data that followed a normal distribution were presented as mean ± standard deviation; one-way analysis of variance (ANOVA) was used to compare the means between multiple groups. The S-N-K method was used to test the comparison between two groups. Abnormal distribution data were described as median (interquartile range) [M (P25–P75)], and the Mann–Whitney *U* test was used for comparison between two groups. Multiple group comparisons were conducted using the Kruskal–Wallis *H* test, followed by the *post-hoc* test. Count data were expressed in frequency, and comparison between groups was performed using the chi-square test or Fisher’s exact test. Spearman correlation analysis was used to investigate the correlation between the CALLY index and WBC counts and protein contents of CSF in patients with ANS. A multiple factor logistic forward stepwise regression model was used to analyze the independently associated factors of ANS, plotting receiver operating characteristic (ROC) curves to analyze the specificity and sensitivity of the CALLY index in predicting ANS and area under the curve (AUC) below the ROC curve to determine the predictive value. The comparison of data before and after treatment in patients with ANS was conducted using paired samples *t*-test (normal distribution data) or Wilcoxon signed rank test (abnormal distribution data). A two-sided test with *p* < 0.05 was used to determine statistical significance.

## Results

### Baseline characteristics in three groups

**Figure 1:**
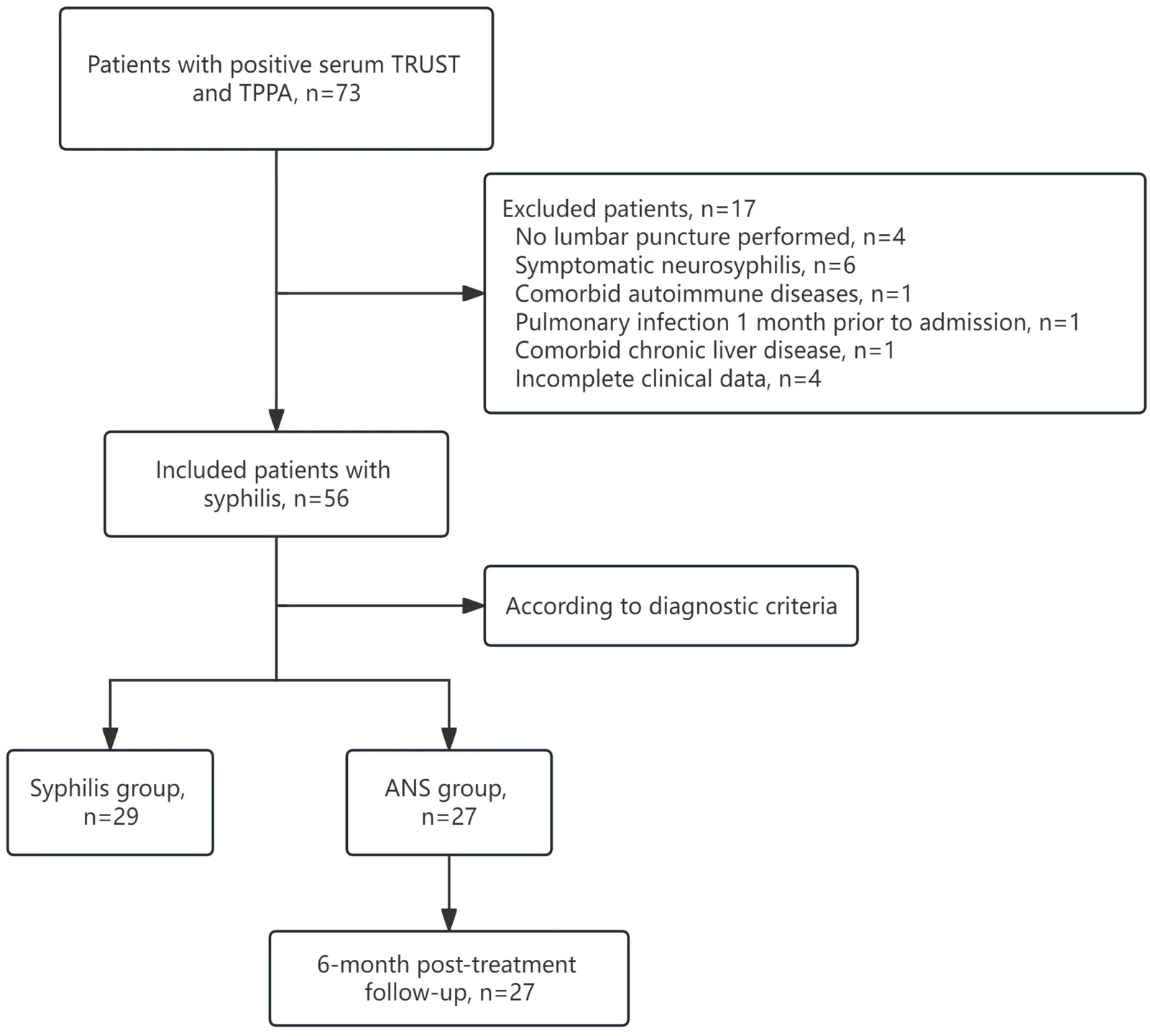
Study flowchart.

Through the hospital electronic medical record system, we found that there were 73 patients with serum TRUST (+) and TPPA (+), and we analyzed their baseline characteristics. Among them, 17 patients did not meet the inclusion criteria. Finally, a total of 56 patients were included for analysis and all were followed up, as shown in the study flowchart (**Figure 1**). Among the 56 patients included, based on the diagnostic criteria for ANS, 27 patients were included in the ANS group, including 24 male and 3 female patients, aged 28–72 years (49.7 ± 12.0 years); BMI range was 19.4–28.4 (24.4 ± 2.5). Among them, there were 4 cases of syphilis stage I, 13 cases of stage II, and 10 cases of stage III; 14 cases of smoking; and 10 cases of drinking; Fourteen cases had been treated with penicillin in the past. Nine cases had comorbidity with hypertension, two cases had diabetes, and one case had coronary heart disease. Twenty-nine patients were in the syphilis group, including 20 male and 9 female patients, aged 19–74 years (43.4 ± 16.5 years); BMI range was 17.4–31.2 (23.5 ± 2.9). Among them, there were 8 cases of syphilis stage I, 14 cases of stage II, and 7 cases of stage III; 13 cases of smoking; and 7 cases of drinking. Seventeen cases had been treated with penicillin in the past. Seven cases had comorbidity with hypertension, three cases had diabetes, and one case had coronary heart disease. The HC group had 32 cases: 25 men and 7 women, aged 27 to 63 years (49.2 ± 7.4 years); BMI range was 19.8–34.5 (24.9 ± 3.3). Among them, there were 12 cases of smoking and 12 cases of drinking. Eleven cases had comorbidity with hypertension, three cases had diabetes, and three cases had coronary heart disease. There was no significant difference among the three groups in age, sex, smoking, drinking, BMI, and comorbidities (hypertension, diabetes, and coronary heart disease) (*p* > 0.05). The WBC counts and protein contents in CSF and the proportion of blood TRUST titers ≥1:16 in the ANS group were significantly higher than those in the syphilis group (*p* < 0.01). There was no significant difference in the course of disease, syphilis stage, and number of previous antisyphilitic treatments between the two groups of patients (*p* > 0.05) (**Table 1**).

**Table 1:** Comparison of baseline characteristics among three groups.

Characteristics	Syphilis group (n = 29)	ANS group (n = 27)	HC group (n = 32)	F/χ^2^/*Z*/*t*	P
Age (years)	43.4 ± 16.5	49.7 ± 12.0	49.2 ± 7.4	2.322	0.1042
Gender, *n* (%)					
Male	20 (69.0%)	24 (88.9%)	25 (78.1%)	3.0808	0.1939
Female	9 (31.0%)	3 (11.1%)	7 (21.9%)		
Comorbidity (*n*)					
Hypertension	7	9	11	1.1779	0.8817
Type 2 diabetes	3	2	3		
Coronary heart disease	1	1	3		
Smoking	13	14	12	1.227	0.5415
Drinking	17	10	12	1.523	0.4670
BMI	23.5 ± 2.9	24.4 ± 2.5	24.9 ± 3.3	1.721	0.1852
CSF					
WBC counts (×10^6^/L)	2.0 (1.0, 3.0)	11.0 (8.0, 40.0)	NA	3.068	0.0034
Protein (mg/dL)	36.6 ± 7.6	63.3 ± 31.6	NA	4.254	0.0001
Chlorine (mmol/L)	126.8 ± 2.8	126.7 ± 2.9	NA	0.1590	0.8743
Glucose (mmol/L)	3.7 ± 0.9	3.7 ± 0.8	NA	0.1555	0.8770
LDH (U/L)	22.0 ± 6.5	24.2 ± 5.2	NA	1.356	0.1809
ADA (U/L)	0.6 ± 0.4	0.7 ± 0.4	NA	0.9453	0.3489
Blood TRUST titers, *n* (%)					
<1:16	24 (82.8%)	13 (48.1%)	NA	7.472	0.0063
≥1:16	5 (17.2%)	14 (51.9%)	NA		
Syphilis stage					
Stage I	8	4	NA	1.831	0.4004
Stage II	14	13	NA		
Stage III	7	10	NA		
Course of disease (months)	12 (7.5, 48)	12 (7, 60)	NA	0.1700	0.8656
Previous antisyphilitic treatment (*n*)	17(58.6%)	14(51.9%)	NA	0.2592	0.6107

HC, healthy control; ANS, asymptomatic neurosyphilis; NA, not applicable; BMI, body mass index; CSF, cerebrospinal fluid; WBC, white blood cell; LDH, lactate dehydrogenase; ADA, adenosine deaminase; TRUST, toluidine red unheated serum test.

### Comparison of immune function/inflammatory response/nutritional status levels among the three groups

There was a statistically significant difference in the percentage of NK cells, neutrophil counts, NLR, PLR, CRP, NPR, SII, CAR, the CALLY index, total protein, albumin, and prealbumin levels in peripheral blood among the three groups (*p* < 0.05, *p* < 0.01). Comparing among the groups, it was found that compared with the syphilis group, the patients of the ANS group had a significant decrease in the percentage of NK cells, IgA, lymphocytes counts, the CALLY index, total protein, and albumin contents (*p* < 0.05, *p* < 0.01). However, neutrophil counts, NLR, PLR, and SII levels were significantly increased (*p* < 0.05, *p* < 0.01). Compared with the HC group, the percentage of NK cells, IgA, the CALLY index, and the contents of total protein and albumin were significantly reduced in patients of the ANS group (*p* < 0.05, *p* < 0.01), while neutrophil counts, NLR, CRP, NRP, CAR, and SII levels were significantly increased (*p* < 0.05, *p* < 0.01) (**Table 2**).

**Table 2:** Comparison of serum parameters among three groups.

Characteristics	Syphilis group (n = 29)	ANS group (n = 27)	HC group (n = 32)	F/H	P
Cellular immune function					
CD3^+^ (×10^6^/L)	1139.6 ± 449.5	1128.5 ± 403.3	1231.0 ± 440.0	0.439	0.6466
CD19^+^ (×10^6^/L)	201.6 ± 107.6	197.7 ± 86.8	242.5 ± 91.6	1.788	0.1753
CD8^+^ (×10^6^/L)	430.4 ± 221.5	398.3 ± 178.1	421.9 ± 167.0	0.159	0.8537
CD4^+^ (×10^6^/L)	610.6 ± 250.3	666.4 ± 288.1	719.5 ± 299.3	0.832	0.4398
CD56^+^ (×10^6^/L)	287.4 ± 115.8	211.2 ± 106.2	265.1 ± 148.5	1.767	0.1788
CD4^+^/CD8^+^	1.6 ± 0.9	1.9 ± 1.0	1.8 ± 0.7	0.441	0.6453
Percentage of T lymphocytes (%)	71.3 ± 5.9	73.7 ± 5.6	70.4 ± 7.5	2.001	0.1415
Percentage of B lymphocytes(%)	13.0 ± 3.6	12.7 ± 3.7	14.1 ± 4.6	1.052	0.3537
Percentage of helper T cells (%)	40.4 ± 8.8	41.7 ± 9.0	40.8 ± 7.5	0.185	0.8316
Percentage of NK cells (%)	14.8 ± 6.6	10.8 ± 4.5^##;^**	15.2 ± 7.1	4.261	0.0172
Humoral immune function					
C3 (g/L)	1.1 ± 0.2	1.1 ± 0.2	1.1 ± 0.2	0.030	0.9709
C4 (g/L)	0.3 ± 0.1	0.4 ± 0.2	0.3 ± 0.1	0.305	0.7380
IgA (g/L)	2.4 ± 0.9	1.8 ± 0.6^##;^**	2.5 ± 0.8	6.277	0.0029
IgG (g/L)	12.2 ± 2.9	12.0 ± 2.9	13.8 ± 3.6	1.533	0.2228
IgM (g/L)	1.1 ± 0.6	0.9 ± 0.5	0.9 ± 0.5	1.101	0.3380
Inflammatory response					
WBC (×10^9^/L)	6.0 ± 1.3	6.7 ± 2.7	5.6 ± 1.2	2.993	0.0555
RBC (×10^12^/L)	4.6 ± 0.6	4.6 ± 0.4	4.8 ± 0.5	0.969	0.3837
Hemoglobin (g/L)	140.5 ± 18.3	138.8 ± 12.7	147.1 ± 14.8	2.426	0.0945
Platelet (×10^9^/L)	213.0 ± 56.5	211.9 ± 62.8	216.9 ± 57.8	0.059	0.9425
Lymphocytes counts (×10^9^/L)	1.9 ± 0.5	1.4 ± 0.5^#^	1.7 ± 0.6	5.603	0.0052
Neutrophils counts (×10^9^/L)	3.4 ± 1.1	4.8 ± 2.7^#.^*	3.2 ± 0.9	7.000	0.0015
NLR	1.8 (1.2, 2.8)	2.8 (2.2, 4.2)^#.^*	1.9 (1.4, 2.4)	17.92	0.0001
PLR	106.5 (90.2,146.6)	142.5 (112.6,205.6)^##^	131.3 (94.1,166.0)	6.480	0.0392
C-reactive protein (mg/L)	0.6 (0.5, 2.6)	1.0 (0.5, 2.15)*	0.5 (0.4, 0.5)	13.69	0.0011
NPR	0.02 (0.01, 0.02)	0.02 (0.02, 0.03)*	0.01 (0.01, 0.02)	8.734	0.0127
SII	346.0 (249.0, 547.8)	552.8 (425.0, 931.8)^##;^**	393.4 (291.4, 480.5)	13.86	0.0010
PAR	5.3 (4.6, 6.0)	5.6 (5.0, 6.5)	4.9 (4.1, 6.8)	1.728	0.4216
CAR	0.01 (0.01, 0.05)**	0.03 (0.01, 0.07)**	0.01 (0.01, 0.02)	25.65	0.0001
CALLY index	11.3 (5.6, 20.0)	5.1 (1.8, 8.3)^##;^**	12.9 (9.4, 17.6)	23.12	0.0001
Nutritional status					
Total protein (g/L)	65.1 ± 5.2	61.3 ± 4.1^#.^ **	66.7 ± 5.1	9.260	0.0002
Albumin (g/L)	39.5 ± 3.7	36.3 ± 3.0^##.^ **	40.6 ± 2.8	13.53	0.0001
Globulin (g/L)	25.6 ± 3.2	25.0 ± 3.7	26.1 ± 3.8	0.712	0.4938
A/G ratios	1.5 ± 0.2	1.5 ± 0.3	1.6 ± 0.3	0.849	0.4319
Prealbumin (mg/L)	269.6 ± 62.8	249.3 ± 55.4*	302.3 ± 55.5	6.268	0.0300

*p < 0.05, **p < 0.01 vs. HC group; ^#^*p* < 0.05, ^##^*p* < 0.01 vs. syphilis group.

HC, healthy control; ANS, asymptomatic neurosyphilis; NK, natural killer; WBC, white blood cell; NLR, neutrophil/lymphocyte ratio; PLR, platelet/lymphocyte ratio; NPR, neutrophil/platelet ratio; SII, systemic immune inflammatory index; PAR, platelet/albumin ratio; CAR, C-reactive protein/albumin ratio; CALLY, C-reactive protein–albumin–lymphocyte.

### Multivariate binary logistic regression analysis of the independently associated factors for ANS in patients with syphilis

Using the presence of concurrent ANS as the dependent variable, a multivariate binary logistic regression analysis was conducted to look for statistically significant factors (*p* < 0.01) (percentage of NK cells, IgA, PLR, SII, the CALLY index, and blood TRUST titer ≥ 1:16) as independent variables in univariate analysis (because the CALLY index shares components with CRP, albumin, and lymphocyte-derived indices, there is a collinearity problem between albumin and the CALLY index, and albumin was not included in the model analysis, although *p* < 0.01 was found in the univariate analysis); we found that the low levels of the CALLY index was an independently associated factor for developing ANS in patients with syphilis (*p* < 0.05), while percentage of NK cells, IgA, PLR, SII, and blood TRUST titers ≥1:16 were not independently associated factors for ANS in patients with syphilis (*p* > 0.05) (**Table 3**).

**Table 3:** Multivariate binary logistic regression analysis of independently associated factors for ANS in patients with syphilis.

Variable	*β*	Wald	*p*	OR	95% CI
PLR	0.009	0.599	0.439	1.009	0.987–1.031
SII	0.002	0.665	0.415	1.002	0.997–1.007
CALLY index	−0.216	6.166	0.013	0.806	0.679–0.955
Percentage of NK cells	−0.156	3.402	0.065	0.855	0.725–1.010
TRUST titers ≥1:16	0.157	2.256	0.133	1.170	0.725–1.010
IgA	−0.191	3.558	0.059	0.826	0.953–1.435

OR, odds ratio; CI, confidence interval; PLR, platelet/lymphocyte ratio; SII, systemic immune inflammatory index; CALLY, C-reactive protein–albumin–lymphocyte; NK, natural killer; TRUST, toluidine red unheated serum test.

### Correlation analysis

**Figure 2:**
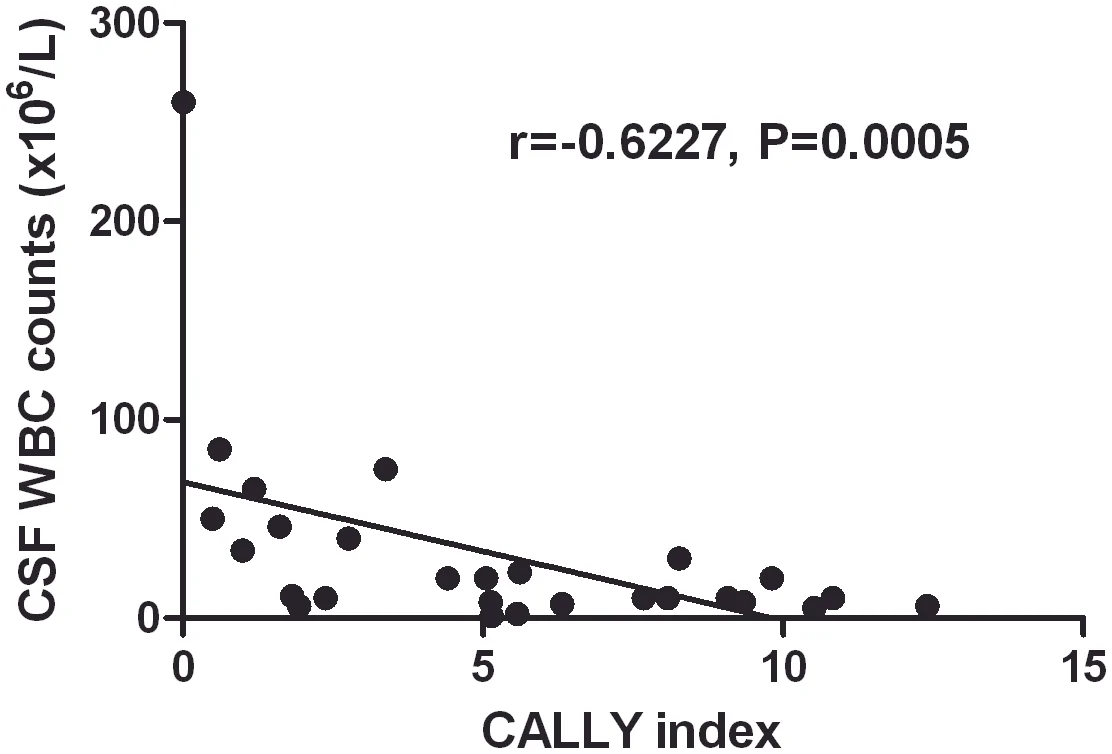
There was a negative correlation between the CALLY index and WBC counts of CSF in patients with ANS (*r* = −0.6227, *p* = 0.0005).

**Figure 3:**
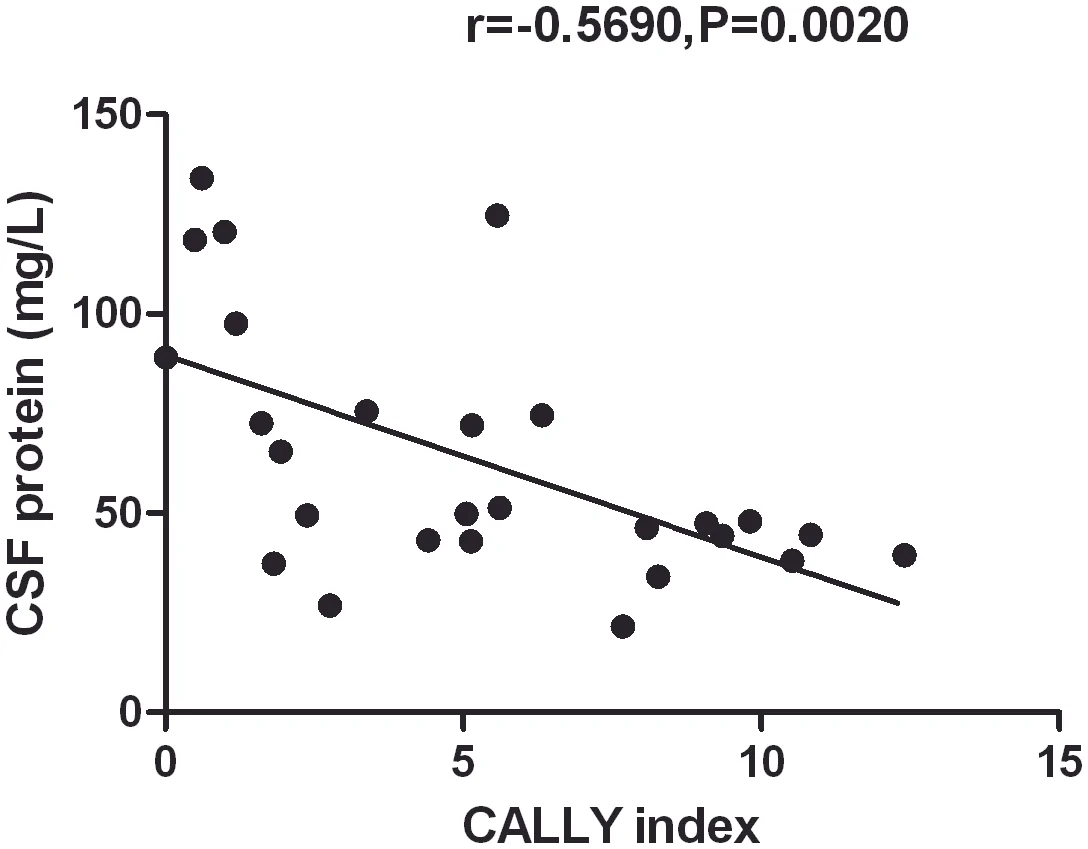
There was a negative correlation between the CALLY index and protein contents of CSF in patients with ANS (*r* = −0.5690, *p* = 0.0020).

Further analysis showed that the CALLY index in patients with ANS was negatively correlated with WBC counts and protein contents of CSF (*r* = −0.6227, *p* = 0.0005; *r* = −0.5690, *p* = 0.0020) (**Figures 2**, **3**).

**Figure 4:**
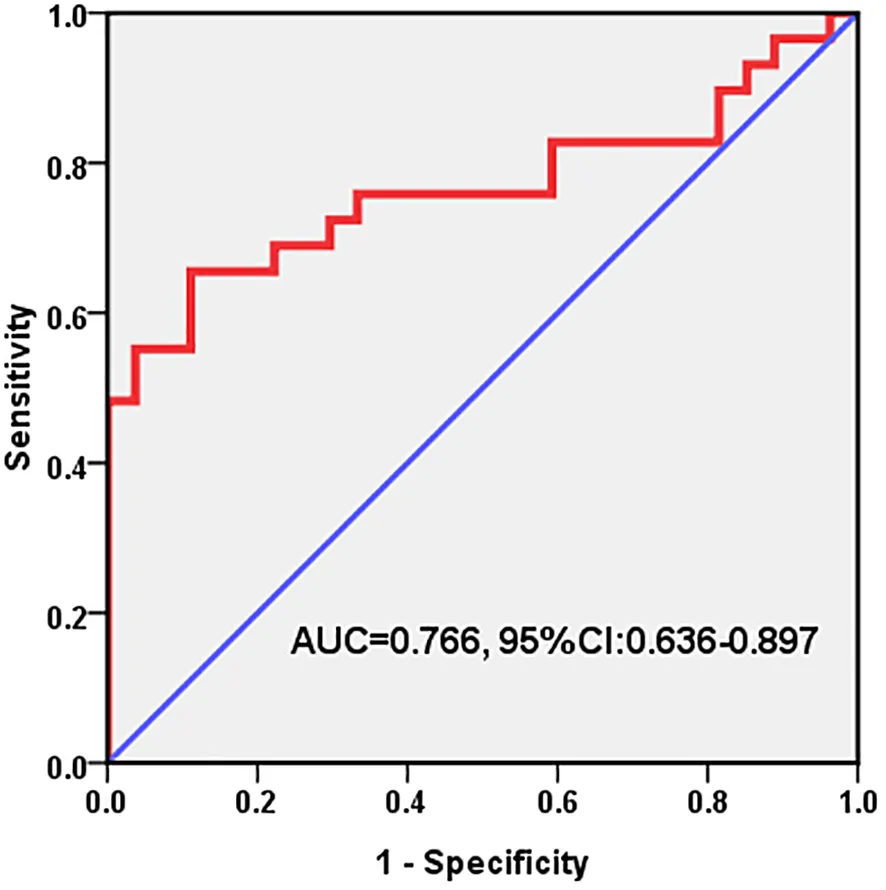
The area under the curve (AUC), sensitivity, and specificity of the CALLY index in predicting the occurrence of ANS in patients with syphilis were 0.766, 65.5%, and 88.9%, respectively.

Comparison of predictive performance: The ROC curve for predicting ANS was established using the CALLY index, which showed that the prediction effect of the CALLY index on ANS occurrence was AUC = 0.766, 95% confidence interval (CI): 0.636–0.897, with a sensitivity and specificity of 65.5% and 88.9%, respectively (**Figure 4**). However, the AUCs of PLR (0.697), SII (0.648), NK percentage (0.686), blood TRUST titers ≥1:16 (0.655), and IgA levels (0.639) were all lower than that of the CALLY index (**Table 4**).

**Table 4:** The value of indicators in predicting ANS in patients with syphilis.

Characteristics	AUC	95% CI	*p*	Optimal cutoff value	Sensitivity	Specificity
PLR	0.697	0.559–0.835	0.012	112.2	77.8%	57.1%
SII	0.648	0.491–0.805	0.072	396.0	50.0%	76.9%
CALLY index	0.766	0.636–0.897	0.001	9.94	65.5%	88.9%
Percentage of NK cells	0.686	0.546–0.826	0.017	15.8	48.3%	85.2%
TRUST titers ≥1:16	0.655	0.509–0.800	0.047	1:16	48.1%	82.8%
IgA	0.639	0.484–0.794	0.096	2.19	53.8%	73.9%

AUC, area under the curve; CI, confidence interval; PLR, platelet/lymphocyte ratio; SII, systemic immune inflammatory index; CALLY, C-reactive protein–albumin–lymphocyte; NK, natural killer; TRUST, toluidine red unheated serum test.

### Changes in immune function/inflammatory response/nutritional status levels of patients with ANS before and 6 months after treatment

Twenty-seven patients with ANS were followed up after high-dose penicillin treatment, and blood and CSF recheck were completed, with the average follow-up time being 6.7 ± 0.9 months (range, 6–10 months). Compared with before treatment, the neutrophil counts, NLR, PLR, SII, the WBC counts, and protein contents in CSF were significantly reduced (*p* < 0.05, *p* < 0.01). However, the percentage of NK cells, the lymphocyte counts, the CALLY index, and A/G ratios were significantly increased (*p* < 0.05, *p* < 0.01). No significant differences were observed in other indicators (*p* > 0.05) (**Table 5**).

**Table 5:** Comparison of serum/CSF markers and blood TRUST titers in 27 patients with ANS between before treatment and 6 months follow-up.

Characteristics	Before treatment	6 months posttreatment	*p*
Cellular immune function			
CD3^+^ (×10^6^/L)	1,128.5 ± 403.3	1,223.5 ± 435.8	0.8262 ^a^
CD19^+^ (×10^6^/L)	197.7 ± 86.8	223.6 ± 120.7	0.4862 ^a^
CD8^+^ (×10^6^/L)	398.3 ± 178.1	450.9 ± 230.2	0.8549 ^a^
CD4^+^ (×10^6^/L)	666.4 ± 288.1	710.2 ± 276.5	0.8137 ^a^
CD56^+^ (×10^6^/L)	211.2 ± 106.2	236.0 ± 105.5	0.2855 ^a^
CD4^+^/CD8^+^	1.9 ± 1.0	1.9 ± 1.0	0.1371 ^a^
Percentage of T lymphocytes (%)	73.7 ± 5.6	71.5 ± 6.3	0.1905 ^a^
Percentage of B lymphocytes (%)	12.7 ± 3.7	13.3 ± 4.4	0.5400 ^a^
Percentage of helper T cells (%)	41.7 ± 9.0	40.4 ± 8.9	0.4275 ^a^
Percentage of NK cells (%)	10.8 ± 4.5	14.7 ± 6.3	0.0063 ^a^
Humoral immune function			
C3 (g/L)	1.1 ± 0.2	1.0 ± 0.2	0.0533 ^a^
C4 (g/L)	0.4 ± 0.2	0.3 ± 0.1	0.0944 ^a^
IgA (g/L)	1.8 ± 0.6	1.9 ± 0.6	0.6411 ^a^
IgG (g/L)	12.0 ± 2.9	11.7 ± 2.4	0.5625 ^a^
IgM (g/L)	0.9 ± 0.5	0.8 ± 0.4	0.8750 ^a^
Inflammatory response			
WBC (×10^9^/L)	6.7 ± 2.7	5.8 ± 1.7	0.0960 ^a^
RBC (×10^12^/L)	4.6 ± 0.4	4.6 ± 0.5	0.4165 ^a^
Hemoglobin (g/L)	138.8 ± 12.7	141.0 ± 11.9	0.2792 ^a^
Platelet (×10^9^/L)	211.9 ± 62.8	205.7 ± 55.1	0.3433 ^a^
Lymphocytes counts (×10^9^/L)	1.4 ± 0.5	1.7 ± 0.5	0.0181 ^a^
Neutrophils counts (×10^9^/L)	4.8 ± 2.7	3.5 ± 1.4	0.0345 ^a^
NLR	2.8 (2.2, 4.2)	1.9 (1.5, 3.0)	0.0297 ^b^
PLR	142.5 (112.6, 205.6)	124.1 (96.9, 149.8)	0.0062 ^b^
C-reactive protein (mg/L)	1.0 (0.5, 2.15)	0.5 (0.5, 1.2)	0.2783 ^b^
NPR	0.02 (0.02, 0.03)	0.02 (0.01, 0.02)	0.0760 ^b^
SII	552.8 (425.0, 931.8)	391.7 (303.7, 556.8)	0.0229 ^b^
PAR	5.6 (5.0, 6.5)	5.8 (5.0, 6.3)	0.3147 ^b^
CAR	0.03 (0.01, 0.07)	0.02 (0.01, 0.03)	0.2912 ^b^
CALLY index	5.1 (1.8, 8.3)	8.7 (4.9, 12.6)	0.0072 ^b^
Nutritional status			
Total protein (g/L)	61.3 ± 4.1	60.7 ± 3.4	0.3171 ^a^
Albumin (g/L)	36.3 ± 3.0	36.5 ± 2.5	0.7087 ^a^
Globulin (g/L)	25.0 ± 3.7	24.2 ± 2.4	0.1438 ^a^
A/G ratios	1.5 ± 0.3	1.6 ± 0.2	0.0294 ^a^
Prealbumin (mg/L)	249.3 ± 55.4	269.4 ± 63.3	0.1758 ^a^
CSF			
WBC counts (× 10^6^/L)	11.0 (8.0, 40.0)	2.0 (1.0, 8.8)	0.0205 ^b^
Protein (mg/dL)	63.3 ± 31.6	50.4 ± 18.7	0.0076 ^a^
Chlorine (mmol/L)	126.7 ± 2.9	127.6 ± 2.8	0.3246 ^a^
Glucose (mmol/L)	3.7 ± 0.8	3.6 ± 0.5	0.4229 ^a^
LDH (U/L)	24.2 ± 5.2	22.8 ± 5.4	0.1917 ^a^
ADA (U/L)	0.7 ± 0.4	0.7 ± 0.4	0.3364 ^a^
Blood TRUST titer, *n* (%)			
<1:16	13 (48.1%)	20 (74.1%)	0.0510 ^c^
≥1:16	14 (51.9%)	7 (25.9%)	

HC, healthy control; ANS, asymptomatic neurosyphilis; NK, natural killer; WBC, white blood cell; NLR, neutrophil/lymphocyte ratio; PLR, platelet/lymphocyte ratio; NPR, neutrophil/platelet ratio; SII, systemic immune inflammatory index; PAR, platelet/albumin ratio; CAR, C-reactive protein/albumin ratio; CALLY, C-reactive protein–albumin–lymphocyte; CSF, cerebrospinal fluid; LDH, lactate dehydrogenase; ADA, adenosine deaminase; TRUST, toluidine red unheated serum test. ^a^Paired samples *t*-test; ^b^Wilcoxon signed-rank test; ^c^Chi-square test.

## Discussion

NS is caused by the invasion of *T. pallidum* into the CNS, leading to lesions in the meninges, cerebrovascular, brain parenchyma, spinal cord, etc. Clinical manifestations can range from no neurological symptoms or signs to complex and diverse neurological damage symptoms. In severe cases, it may result in irreversible organ dysfunction or even death (7–9). NS is a serious complication of syphilis, but its pathogenesis has not been fully elucidated. The relationship between the host’s immune function, inflammatory response, and immune regulation is considered to play an important role in this process (21, 44–46). This study analyzed the markers of immune cells, inflammatory response, and nutritional status in peripheral blood of HIV-negative patients with ANS, revealing significant immune function–inflammatory response–nutritional status dysfunctions in patients with ANS.

NK cells are an important component of the innate immune system and play a crucial anti-infective role, which can regulate adaptive immunity by directly killing infected cells and secreting cytokines (47–49). Previous studies (50, 51) have shown that a decrease or impaired function of NK cells may weaken the host’s ability to clear *T. pallidum*, allowing pathogens to persist in the CNS. Our results are consistent with this. This study found that, compared with the syphilis group and the HC group, the percentage of NK cells in patients with ANS was significantly reduced. In terms of humoral immunity, the serum IgA levels of patients with ANS were significantly reduced, while there were no significant differences in IgG, IgM, and complement C3 and C4 levels among these three groups. IgA is the main antibody in mucosal immunity and plays an important role in defending against pathogen invasion (52, 53). The decrease in IgA levels may facilitate the breakthrough of *T. pallidum* from peripheral blood through the blood–brain barrier into the CNS. However, there was no synchronous decrease in IgG and IgM levels in patients with ANS, suggesting that their systemic humoral immunity may still be relatively intact, but there may be selective defects in local or specific types of immunoglobulins.

In addition to immune dysfunction, another finding of this study is that patients with ANS exhibit significant inflammation–nutrient imbalance. The coexistence pattern of high inflammation and low nutritional levels suggests that patients with ANS may be in a chronic low-grade inflammatory state, which not only depletes the body’s nutritional reserves, but also may damage the blood–brain barrier through the release of inflammatory mediators, thereby promoting the colonization and immune escape of *T. pallidum* in the CNS.

Based on the different clinical characteristics, NS is divided into the asymptomatic and the symptomatic type (syphilitic meningitis, meningovascular NS, general paresis, tabes dorsalis, syphilitic gummas, etc.) (3, 5). Symptomatic NS, because of its clinical manifestations, deserves sufficient attention from clinicians, patients, and their families. However, ANS, as a special type, with the absence of neurological symptoms and signs and the presence of abnormal CSF laboratory tests as its main features, is easily overlooked and missed due to its lack of clinical symptoms. Patients with ANS can be completely cured after early standardized treatment. Therefore, in the clinical real world, clinicians should pay more attention to early diagnosis and treatment. Although previous studies have found that some CSF/blood markers are helpful for the diagnosis of NS, most of them are targeted in patients with symptomatic NS (17–20). The CALLY index is a complex biomarker that integrates inflammation, nutrition, and immune status. The index can comprehensively reflect the patient’s nutritional status, inflammation level, and immune response, and has been applied in the prognostic evaluation and severity of various diseases (38, 54–57). This study applied the CALLY index to the research of ANS and found it to be potentially associated with the early diagnosis of ANS. However, single indicators reflecting the patient’s immune function, inflammatory response, and nutritional status (such as PLR, SII, percentage of NK cells, TRUST titer, and IgA levels) are not independently associated factors of concurrent ANS. The AUC curve of the CALLY index predicting ANS was 0.766, with a sensitivity and specificity of 65.5% and 88.9%, respectively. This indicates that the CALLY index has good predictive power in distinguishing ANS from patients with simple syphilis. Further correlation analysis showed that the CALLY index of patients with ANS was negatively correlated with WBC counts and protein contents of CSF. Given that WBC counts and protein levels of CSF are important indicators reflecting CNS inflammation and blood–brain barrier disruption, this result further supports the CALLY index as a potential biomarker for assessing the severity of ANS.

In order to clarify whether the imbalance of immune function, inflammatory response, and nutritional status in patients with ANS is reversible, we further analyzed the changes in various indicators of 27 patients with ANS after 6 months of treatment. The results showed that after standardized treatment, the patient’s inflammatory biomarkers are significantly reduced. It is worth noting that the percentage of NK cells and the CALLY index were significantly increased after treatment. These results indicate that the immune inflammatory dysfunctions present in patients with ANS are not irreversible; it can correct this imbalance through effective treatment. This further suggests that early diagnosis and timely intervention are crucial for the immune recovery and prognosis improvement of patients with ANS. Of course, in the real world, some external factors may have an impact on the levels of serum biomarkers, and a more rigorous study needs to be designed to avoid these influencing factors in the future.

This study also has some limitations. Firstly, the sample size is relatively small and this is a single-center retrospective study, which may lead to patient selection bias. This is mainly because we strictly included patients according to the criteria and excluded some patients with incomplete data. [Although the sample size is small, we conducted *post-hoc* power analysis using G*Power3.1 software. The total number of patients in the three groups was 88; when selecting effect size = 0.35 and α = 0.05 (two tailed), we obtained power = 0.83 (>0.8), which indirectly indicates the reliability of our results.] Secondly, this study only observed changes in immune–inflammatory–nutritional indicators in patients with ANS after 6 months of treatment, and longer-term follow-up data could be more valuable. Meanwhile, the predictive performance of the CALLY index still needs to be validated through future multicenter studies with larger sample sizes. Thirdly, this study only explored the changes in peripheral blood indicators and failed to synchronously analyze the immune microenvironment indicators reflecting the CNS, and the correlation between them and peripheral immunity needs further exploration. Fourthly, we classified patients as having ANS primarily on the basis of relatively nonspecific CSF abnormality. Although TRUST (+) and TPPA (+) in CSF have high specificity for the diagnosis of NS, there is still some gap compared to CSF-VDRL. Future research needs to avoid these limitations.

In summary, this study indicates that although patients with ANS have no obvious neurological clinical symptoms, they exhibit peripheral immune dysfunction, enhanced systemic inflammatory response, and worsening nutritional status. This “immune–inflammation–nutrition” triple imbalance state constitutes an important pathophysiological feature of ANS. The CALLY index, as a comprehensive indicator that integrates immune, inflammatory, and nutritional dimensions, has shown preliminary discriminatory ability for the early diagnosis of ANS and indicates its potential as a diagnostic biomarker. At the same time, the early diagnosis and intervention was important. The findings of this study provide references for clinicians to further understand the immune function changes in patients with ANS, optimize screening strategies, and achieve early diagnosis and treatment. Because of the small sample size of this study, large-scale, multicenter, prospective cohort studies are needed to further confirm the results.

## Data Availability

The raw data supporting the conclusions of this article will be made available by the authors, without undue reservation.
